# Supplementary material for: Transmission-clearance trade-offs indicate that dengue virulence evolution depends on epidemiological context
Source: Nat Commun. 2018 Jun 15;9:2355. doi: 10.1038/s41467-018-04595-w (PMC6003961; doi:10.1038/s41467-018-04595-w)
Supplement: Supplementary file 1 — Supplementary Information [file 41467_2018_4595_MOESM1_ESM.pdf]

Transmission-clearance trade-offs indicate that dengue virulence evolution depends on epidemiological context
Supplementary Material

Rotem Ben-Shachar[1,2,3,4*], Katia Koelle [2*,5]

1 Program in Computational Biology and Bioinformatics, Duke University, Durham, NC, 27708, USA

2 Department of Biology, Duke University, Durham, NC, 27708, USA

3 Department of Integrative Biology, University of California Berkeley, CA, 94720, USA

4 Division of Infectious Diseases and Vaccinology, School of Public Health, University of California, Berkeley, 94720, USA

5 Department of Biology, Emory University, Atlanta, GA, 30322, USA

* rbenshachar@gmail.com, katia.koelle@emory.edu

Supplementary Table 1: Parameters of the within-host model fit to viremia measurements. If the parameter is estimated, 95% confidence intervals are shown in parentheses. Values for the model fit to both the full dataset and the data subset are provided.

Parameter	Description	Units	Full dataset value	Subset dataset value
Estimated				
β_{PI}	viral infectivity rate (1°)	(genome copies/ml)$^{-1}$ day^{-1}	$3.99(3.82, 4.16) \times 10^{-10}$	3.11 (2.95, 3.29)$\times10^{-10}$
β_{SI}	viral infectivity rate (2°)	(genome copies/ml)$^{-1}$ day^{-1}	$5.08(4.71, 5.47) \times 10^{-10}$	3.95 (3.74, 4.18)$\times10^{-10}$
q_{PI}	innate IR activate rate (1°)	day^{-1}	$2.1(1.5, 2.9) \times 10^{-3}$	0.23 (0.11, 0.42)
d_N	NK cell decay rate	day^{-1}	0.07 (0.04, 0.1)	0 (assigned)
q_T	T-cell activation rate	(cells/ml)$^{-1}$ day^{-1}	$4.5(4.4, 4.6) \times 10^{-7}$	$4.84(3.38, 6.83) \times 10^{-5}$
c	viral infectivity rate CV	unitless	$0.19(0.16, 0.23)$	0.19 (assigned)
Assigned				
κ	viral clearance rate	day^{-1}	5	5
ω	viral production rate	(genome copies/cell) day^{-1}	10^4	10^4
q_{SI}	innate IR activate rate (2°)	day^{-1}	0	0
α	innate IR clearance rate	day^{-1}	0.001	0.001
δ_T	T-cell clearance rate	day^{-1}	10^{-6}	10^{-6}
IP_g	incubation period	days	5.9	5.9
σ	incubation period sd	log(days)	0.2	0.2
σ_ϵ	measurement error sd	log genome copies/ml	0.5	0.5
Initial conditions				
X_0	uninfected cells	cells/ml	10^7	10^7
Y_0	infected cells	cells/ml	0	0
V_0	viral load	genome copies/ml	10^{-3}	10^{-3}
N_0	NK cells	cells/ml	0	0
T_0	T-cells	cells/ml	0	10^5

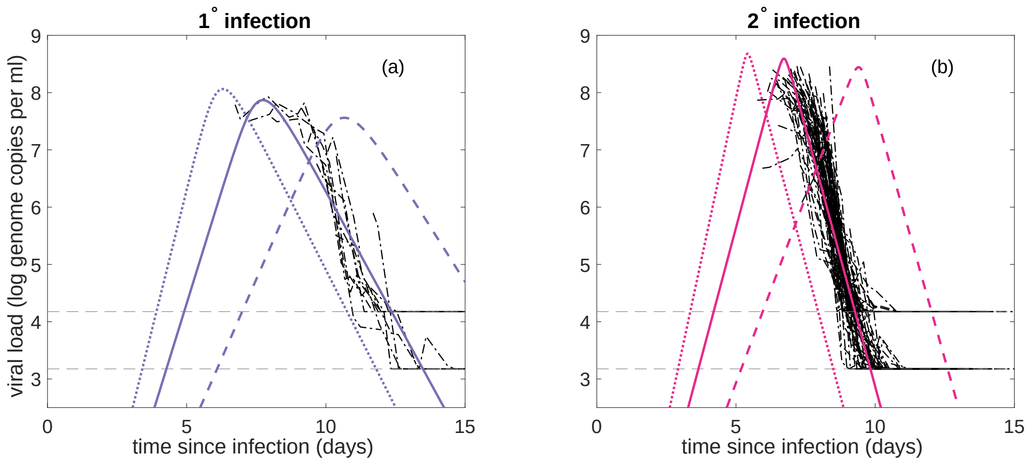

Supplementary Figure 1. Within-host dynamics by immune status for the within-host model fit to the data subset. Model-simulated viral load dynamics alongside viremia measurements from individuals experiencing (a) a primary dengue infection with maximum viral load of ≤ 8 log genome copies/ml and from individuals experiencing (b) a secondary dengue infection with maximum viral load of ≤ 8.5 log genome copies/ml. Solid colored lines show viral load dynamics parameterized with the mean estimated viral infectivity rate. Dotted lines show viral load dynamics parameterized with a viral infectivity rate two standard deviations above the estimated mean. Dashed lines show viral load dynamics parameterized with a viral infectivity rate two standard deviations below the estimated mean. Viremia measurements are shown in black and are shifted in time based on inferred individual incubation periods using model simulations with mean estimated viral infectivity rates. Viral limits of detection are shown with horizontal gray lines. Parameter values are provided in Supplementary Table 1.

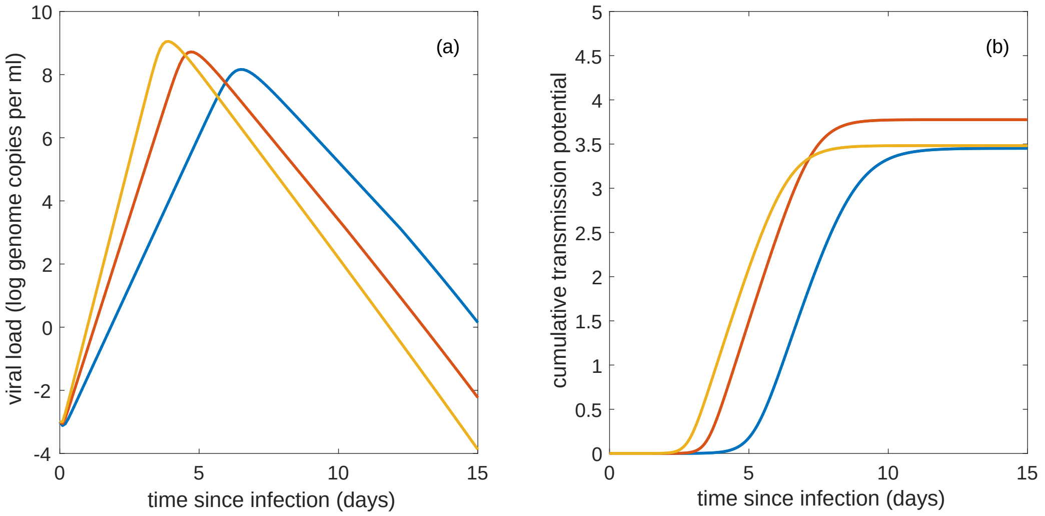

Supplementary Figure 2. Simulated dengue viral load dynamics and accumulation of transmission potential simulated under model parameterizations with different viral production rates.(a) Primary infection viral load dynamics. (b) Primary infection transmission potential accumulation over time since infection. In both subplots, a simulation parameterized with the optimal viral production rate value is shown in red ($\omega = 2.33 \times 10^4$ genome copies per cell per day). A simulation parameterized with a production rate value below the optimal is shown in blue ($\omega = 1.33 \times 10^4$ genome copies per cell per day). A simulation parameterized with a viral production rate value above the optimal one is shown in yellow ($\omega = 3.33 \times 10^4$ genome copies per cell per day). All simulations were parameterized using the mean viral infectivity rate β_{PI} for the model fit to the data subset. All other parameters values are set to the data subset parameter estimates provided in Supplementary Table 1.

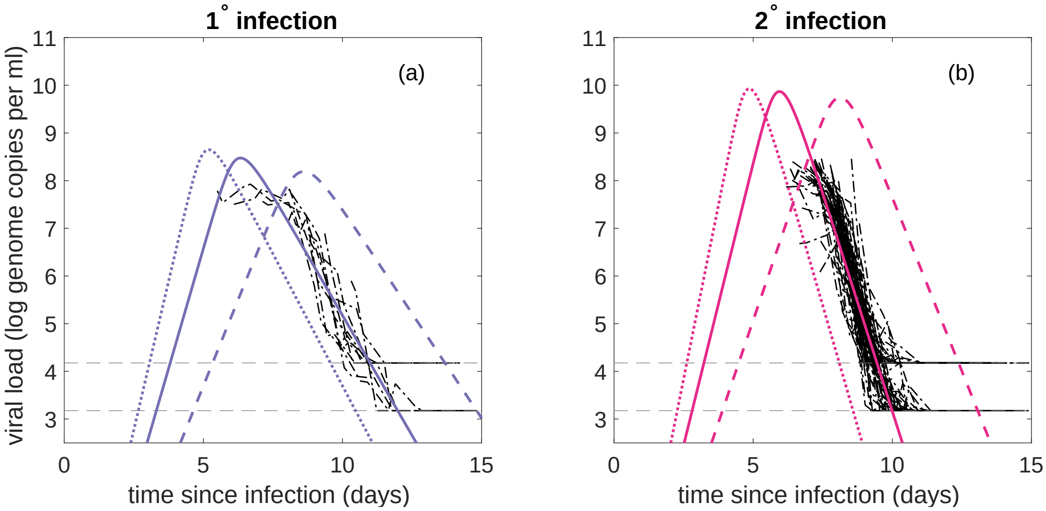

Supplementary Figure 3. Within-host dynamics by immune status for the within-host model fit to the data subset when we assume secondary infections are cleared entirely by the innate immune response. Model-simulated viral load dynamics alongside viremia measurements from individuals experiencing (a) a primary dengue infection with maximum viral load of ≤ 8 log genome copies/ml and from individuals experiencing (b) a secondary dengue infection with maximum viral load of ≤ 8.5 log genome copies/ml. Solid colored lines show viral load dynamics parameterized with the mean estimated viral infectivity rate. Dotted lines show viral load dynamics parameterized with a viral infectivity rate two standard deviations above the estimated mean. Dashed lines show viral load dynamics parameterized with a viral infectivity rate two standard deviations below the estimated mean. Viremia measurements are shown in black and are shifted in time based on inferred individual incubation periods using model simulations with mean estimated viral infectivity rates. Viral limits of detection are shown with horizontal gray lines. We fit β_{PI}, q_{PI} and q_{SI}. We set the coefficient of variation to the value provided in Supplementary Table 1 and set β_{SI} based on the ratio of β_{SI}/β_{PI} provided in Supplementary Table 1 and the estimate of β_{PI}. Estimated parameter values and 95% confidence intervals are: $\beta_{PI} = 4.5 \times 10^{-10}$ $(4.3 \times 10^{-10}, 4.7 \times 10^{-10})$ (genome copies/ml)$^{-1}$ day^{-1}, $\beta_{SI} = 5.7 \times 10^{-10}$ $(5.4 \times 10^{-10}, 6.0 \times 10^{-10})$ (genome copies/ml)$^{-1}$ day^{-1}, $q_{PI} = 0.09$ (.03, 0.22)/day, $q_{SI} = 1.8 \times 10^{-3}$ $(1.3 \times 10^{-3}, 2.5 \times 10^{-3})$ /day. Again, $d_N = 0$.

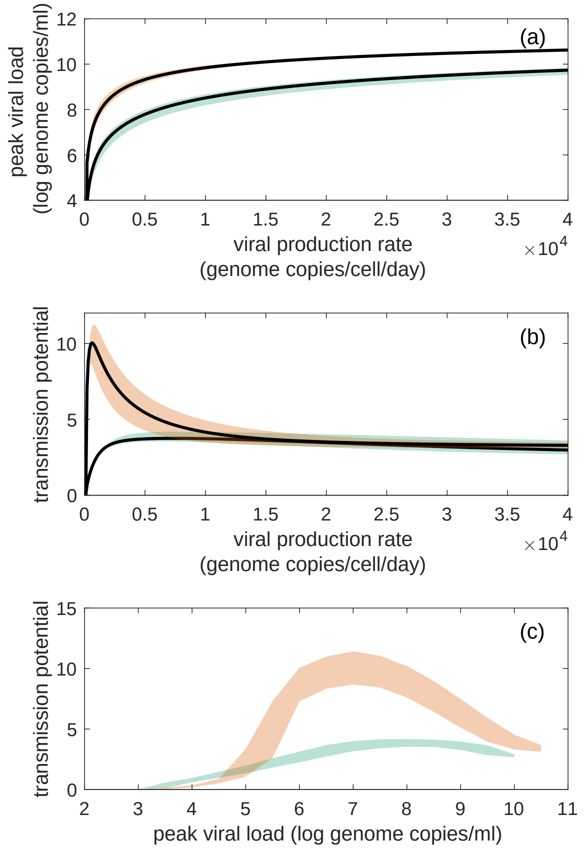

Supplementary Figure 4. Model-predicted transmission-virulence trade-offs for dengue virus when we assume secondary infections are cleared entirely by the innate immune response. (a) Model-predicted relationship between the viral production rate and peak viral load. (b) Model-predicted relationship between the viral production rate and dengue's transmission potential. (c) Model-predicted relationship between peak viral load and dengue's transmission potential. In (a-c), shaded regions show uncertainty estimates derived from variation in the viral infectivity rate (Methods). Primary infections are shown in green and secondary infections are shown in orange.

Supplementary Note 1: Fitness trade-offs during secondary infections for an alternative T-cell formulation

We consider here an alternative T-cell formulation in which the rate of T-cell production saturates at high antigen levels. The alternative T-cell parameterization is given by: $dT/dt = \frac{q_{T_a} Y}{Y+a} T$, where a is the level of infected cells in which T-cell activation reaches half of its maximal value. We were unable to simultaneously estimate the value of a and the other model parameters that we estimated when fitting Equations 1 to the data subset. We therefore set the value of a to different values and fit this alternative T-cell model to the subset data, estimating parameters β_{PI}, q_{PI} and q_{T_a} for each value of a considered. As in Supplementary Figure 1, we set d_N to 0, q_{SI} to 0, c to 0.19, and let β_{SI} be the product of β_{PI} and the ratio of β_{SI}/β_{PI} from the full dataset fit. Simulations of secondary infection viral load dynamics under this T-cell model, using the mean viral infectivity rate β_{SI}, are shown in Supplementary Figure 5a. Supplementary Figure 5b shows that peak viral load increases with the viral production rate, similar to what was observed in Fig. 3a. Supplementary Figure 5c shows that the transmission potential again peaks at intermediate viral production rates, corresponding to intermediate virulence. The value of the viral production rate that maximizes transmission potential is relatively insensitive to the extent to which T-cell activation rates saturate with increasing antigen levels.

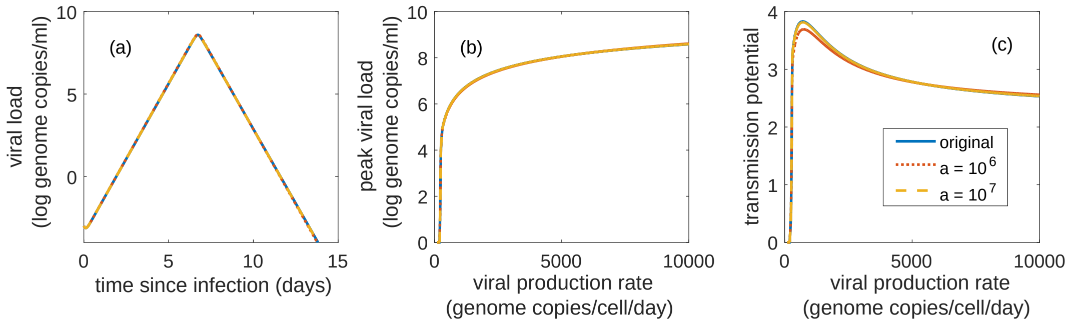

Supplementary Figure 5. Virulence trade-offs in secondary dengue infections for an alternative T-cell formulation. (a) Simulated viral load dynamics for the alternative T-cell model parameterized with different values of a. (b) Model-predicted relationship between the viral production rate and peak viral load. (c) Model-predicted relationship between the viral production rate and dengue's transmission potential. The original T-cell formulation ("original") is the one used in Fig. 3: $dT/dt = q_T Y T$. Estimated parameter values and 95% confidence intervals are: $a = 10^6$ cells/ml: $\beta_{SI} = 4.0 \times 10^{-10}$ $(3.8 \times 10^{-10}, 4.2 \times 10^{-10})$ (genome copies/ml)$^{-1}$ day^{-1}, $q_{T_a} = 53$ (38, 72) per day; $a = 10^7$ cells/ml: $\beta_{SI} = 4.0 \times 10^{-10}$ $(3.7 \times 10^{-10}, 4.2 \times 10^{-10})$ (genome copies/ml)$^{-1}$ day^{-1}, $q_{T_a} = 488$ (342, 686) per day. Again, $d_N = 0$.

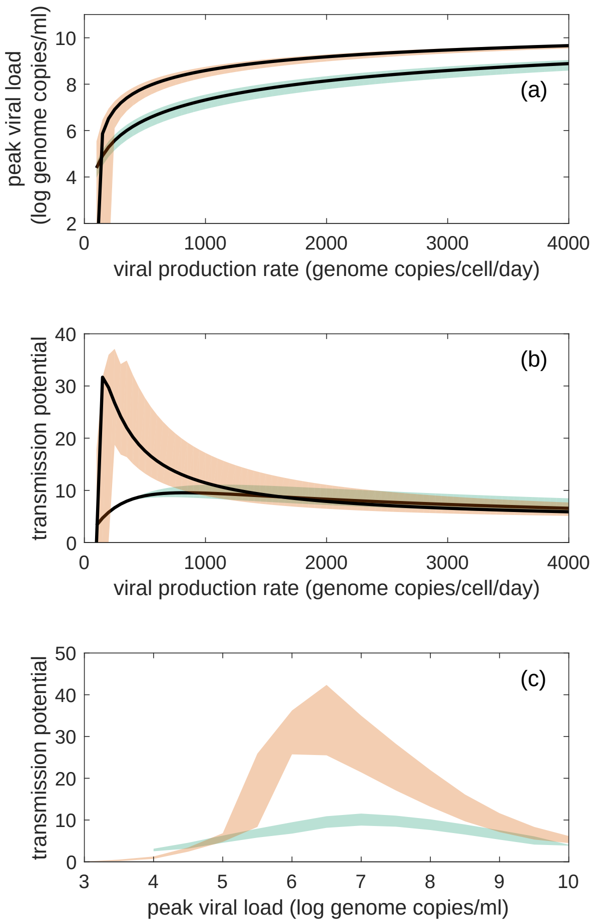

Supplementary Figure 6. Fitness trade-offs during dengue infections, stratified by immune status for the within-host model parameterized to the full dataset. (a) Model-predicted relationship between the viral production rate and peak viral load. (b) Model-predicted relationship between the viral production rate and dengue's transmission potential. (c) Model-predicted relationship between peak viral load and dengue's transmission potential. In (a-c), shaded regions show uncertainty estimates, derived from variation in the viral infectivity rate (Methods). Primary infections are shown in green and secondary infections are shown in orange. At low viral production rates, primary dengue infection simulations unrealistically had viral load levels returning from low levels. Instead of explicitly incorporating the adaptive immune response to ultimately clear primary dengue infections, we set the innate immune response decay rate d_N to 0 in these simulations to more simply capture the effect that the adaptive immune response would have in clearing the viral infection. Primary infection simulations with d_N set to 0 instead of to 0.07 had little effect on viral load dynamics at $\omega = 10^4$ genome copies/cell/day.

Supplementary Note 2: Fitness trade-offs for serotypes other than DENV-1

We here consider selection on viral production rates for dengue infections with serotypes other than DENV-1. Because we do not consider serotype-specific differences in within-host dynamics, these differences arise solely from serotype-specific differences in the probability of viral transmission from infected human to susceptible mosquito. Nguyen et al. showed that the probability of viral transmission from human to mosquito strongly depends on the viral load of the infected human, with some quantitative differences between the serotypes [1] (Supplementary Figure 7a).

For any given serotype, dengue's transmission potential is maximized at intermediate viral production rates in both primary and secondary infections, with secondary infections having their transmission potential maximized at lower viral production rates than primary infections (Supplementary Figure 7b). Dengue's transmission potential is also maximized at intermediate virulence (Supplementary Figure 7c), as is expected from the monotonically increasing relationship between the viral production rate and peak viral load (Fig. 3a).

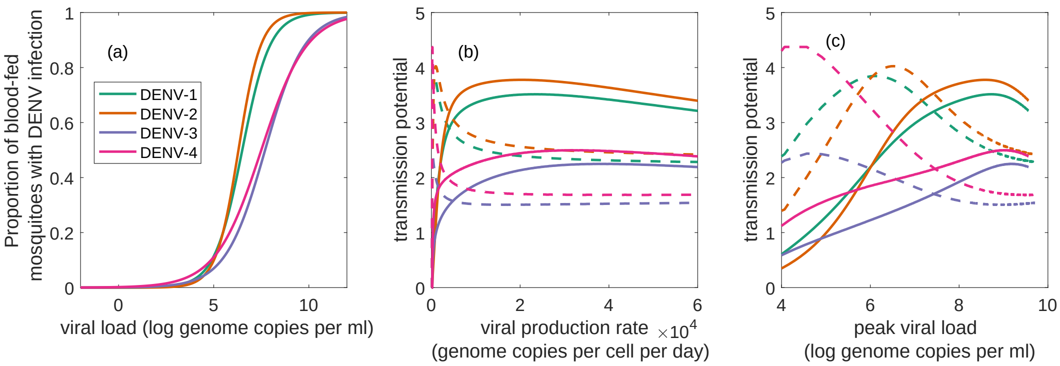

Supplementary Figure 7. Fitness trade-off results are robust to dengue serotype used for mapping viral load to probability of mosquito infection. (a) The estimated relationship between host viral load V and the probability of transmitting dengue to the mosquito vector p reproduced from [1] for each of the four DENV serotypes. (b) Model-predicted relationship between the viral production rate and dengue's transmission potential. (c) Model-predicted relationship between peak viral load and dengue's transmission potential. In (b, c), solid lines show primary infections and dashed lines show secondary infections. All models are parameterized with mean viral infectivity rates and parameters estimated from the fit of Equations 1 to the HCMC data subset.

Supplementary Note 3: Quantifying population-level viral transmission potential when viral production rates further impact mosquito-to-human transmission success

Recall from equation 3 in the main text that the basic reproduction number for a vector-borne disease is given by:

$$R_0 = mb^2 p_{hm} \int_0^\infty p(V(\tau)) d\tau$$

where the integral component of this equation is the transmission potential, m is the ratio of female mosquitoes to human hosts, b is the biting rate, and p_{hm} is the probability that an infected mosquito infects a susceptible host from a single bite. In the main text, we assumed that neither m, b, nor p_{hm} were dependent on the viral production rate. Yet, several studies indicate that more virulent dengue genotypes (with higher viral production rates) may transmit more readily from infected mosquito to susceptible host (see Discussion section). We can incorporate this potential relationship between viral production rate and fitness in the mosquito by letting p_{hm} depend positively on the viral production rate ω. To determine the effect of such a positive relationship on the evolution of dengue virulence, we arbitrarily parameterized a logistic function to yield a relationship between viral production rate and p_{hm} (Supplementary Figure 8a). In Supplementary Figure 8b, we show the product of p_{hm} and the transmission potential over a range of viral production rates. This product reflects variation in R_0 between dengue strains differing in viral production rates.

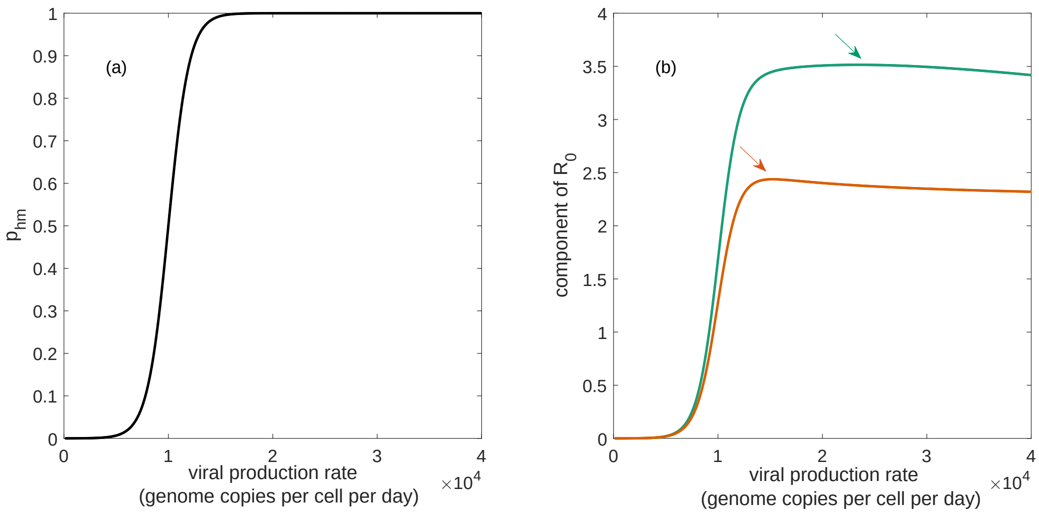

Supplementary Figure 8. Incorporating the effect of the viral production rate on mosquito-to-human transmission success. (a) An assumed relationship between the dengue virus production rate and the probability of an infected mosquito transmitting the virus to a susceptible host, p_{hm}. The logistic model $p_{hm} = \frac{1}{1+e^{-(10+.001\omega)}}$ was used to model this relationship. (b) Model-predicted relationship between the dengue virus production rate and the product of p_{hm} and dengue's transmission potential. As in Figure 3b, green and orange arrows point to the peaks in transmission fitness for primary and secondary infections, respectively.

Supplementary References

1. Nguyen MN, Kien DTHK, Tuan TV, Quyen NTH, Tran CNB, Long VT, et al. Host and viral features of human dengue cases shape the population of infected and infectious Aedes aegypti mosquitoes. Proc Natl Acad Sci U S A. 2013;110(22):9072–7. doi:10.1073/pnas.1303395110.
